# A Comparison of Fosaprepitant and Ondansetron for Preventing Postoperative Nausea and Vomiting in Moderate to High Risk Patients: A Retrospective Database Analysis

**DOI:** 10.1155/2017/5703528

**Published:** 2017-12-19

**Authors:** Chiaki Murakami, Nami Kakuta, Katsuyoshi Kume, Yoko Sakai, Asuka Kasai, Takuro Oyama, Katsuya Tanaka, Yasuo M. Tsutsumi

**Affiliations:** Department of Anesthesiology, Tokushima University, 3-18-15 Kuramoto, Tokushima 770-8503, Japan

## Abstract

Postoperative nausea and vomiting (PONV) occur in 30–50% of patients undergoing general anesthesia and in 70–80% of high PONV risk patients. In this study, we investigated the efficacy of fosaprepitant, a neurokinin-1 (NK1) receptor antagonist, compared to ondansetron, a selective 5-hydroxytryptamine type 3 (5-HT3) receptor antagonist, in moderate to high PONV risk patients from our previous randomized controlled trials. Patients (171 patients from 4 pooled studies) with the Apfel simplified score ≥ 2 and undergoing general anesthesia were randomly allocated to receive intravenous fosaprepitant 150 mg (NK1 group, *n* = 82) and intravenous ondansetron 4 mg (ONS group, *n* = 89) before induction of anesthesia. Incidence of vomiting was significantly lower in the NK1 group compared to the ONS group 0–2, 0–24, and 0–48 hours after surgery (2 versus 17%, 2 versus 28%, and 2 versus 29%, resp.). However, no significant differences in PONV, complete response, rescue antiemetic use, and nausea score were observed between groups 0–48 hours after surgery. In moderate to high PONV risk patients, fosaprepitant decreased the incidence of vomiting and was superior to ondansetron in preventing postoperative vomiting 0–48 hours after surgery.

## 1. Introduction

Postoperative nausea and vomiting (PONV) are distressing and serious adverse effects of anesthesia and surgery. PONV is experienced by 30–50% of patients undergoing general anesthesia and up to 70–80% of high PONV risk patients (female patients, nonsmokers, patients with history of PONV and/or motion sickness, and receiving opioids postoperatively [1–4]). In the “Consensus guidelines for the management of postoperative nausea and vomiting” [4], use of antiemetics for PONV prophylaxis is recommended in moderate to high risk patients. Additionally, in patients receiving prophylactic treatment for PONV, such as ondansetron, a selective 5-hydroxytryptamine type 3 (5-HT3) receptor antagonist, PONV, occurs in up to 30–40% of the patients during the first postoperative day [2, 5, 6]. In addition to patient distress and discomfort, PONV increases the risk of venous thrombus or pulmonary embolism, interferes with physical therapy, and prolongs hospital stay [7–9]. Therefore, more effective therapies for preventing PONV are required, particularly in high risk patients.

Fosaprepitant is a water-soluble phosphorylated prodrug of aprepitant, a highly selective neurokinin-1 (NK1) receptor antagonist, and is rapidly transformed into aprepitant when administered intravenously [10]. Aprepitant blocks NK1 receptors and demonstrates antiemetic efficacy and a long half-life, with two large trials finding aprepitant advantageous in preventing PONV, compared to ondansetron [11–13]. Fosaprepitant is used for managing chemotherapy-induced nausea and vomiting (CINV) and was reported to have superior, longer-lasting effect in preventing CINV, compared to other antiemetics [14].

Recently, we reported that fosaprepitant was more effective than ondansetron in preventing vomiting 0–24 and 0–48 h after surgery [15–17]. In this study, we collected data from our previous studies and evaluated the efficacy of fosaprepitant in preventing PONV compared to ondansetron in moderate to high PONV risk patients.

## 2. Methods

This analysis was based on data from four identically designed, double-blind, randomized, controlled studies. These studies were approved by the Human Research Ethics Committee of the University of Tokushima (Tokushima, Japan) and registered in a clinical trials database (UMIN000008621, 000012999, 000007613, and 000018532). Written informed consent was obtained from all patients, and the studies were conducted in accordance with the principles outlined in the Declaration of Helsinki.

Patients (aged 20–80 years) with the American Society of Anesthesiologists (ASA) physical status of I–III and undergoing general anesthesia were enrolled in the studies. They all had Apfel simplified score for PONV ≥ 2, which includes 4 factors: female sex, history of PONV and/or motion sickness, nonsmoker smoking status, and postoperative opioids use. Patients with 0-1, 2, and 3-4 factors are considered to be at low, moderate, and high risk for PONV, respectively [2]. Exclusion criteria were ASA physical status of IV, neural diseases, abnormal liver and/or renal function, and use of other antiemetics. Patient data recorded included gender, history of PONV and motion sickness, and smoking status.

Patients were randomized and allocated to groups using Quickcalcs software (Graph-Pad Inc., La Jolla, CA, USA). To ensure investigator blinding randomization schedule was generated by a statistician who was not involved in the clinical study. On the day of surgery, patients were randomized to the NK1 group, which received intravenous fosaprepitant (150 mg) and the ONS group, which received intravenous ondansetron (4 mg). Antiemetics were infused over 30 min by anesthesiologists before induction of anesthesia, following approved administration protocols.

Anesthesia was induced with remifentanil 0.3–0.5 *μ*g/kg/min, propofol 1-2 mg/kg, and rocuronium 0.8 mg/kg to facilitate endotracheal intubation. Anesthesia was maintained with propofol (target controlled infusion: 2–4 *μ*g/ml) or volatile anesthetics (sevoflurane 1-2% or desflurane 3-4%) in oxygen with air mixture, remifentanil, and fentanyl. Incremental doses of rocuronium were used as necessary for muscle relaxation, which was reversed by sugammadex 2 mg/kg at the end of surgery. A rescue antiemetic (10 mg metoclopramide) and/or analgesic were administered on patient request.

The incidence of nausea and vomiting, complete response rate (no vomiting and no rescue antiemetic use), rescue antiemetic use, and severity of nausea were evaluated by blinded observers 2, 24, and 48 hours after surgery. The severity of nausea was estimated using the nausea score (0, absence of nausea; 1–3 mild, moderate, and severe nausea, resp.). Scores were evaluated by blinded observers, who determined the highest scores in each surveyed period and all adverse events during the first 48 hours after surgery were recorded.

Statistical analyses were performed using Statistical Package for Social Sciences (SPSS) software (version 22, SPSS INC., Chicago, IL, USA). Data are expressed as means ± standard deviations. *P* values < 0.05 were considered statistically significant without any adjustment for multiplicity of testing. *T*-test and *χ*^2^ test were used for analyzing patient demographics, cumulative incidence of vomiting at each time point, incidence of nausea, rescue antiemetic use, and complete response (no vomiting and no rescue) 0–2, 0–24, and 0–48 hours after surgery. The Mann–Whitney *U* test was used to analyze nausea score at these time points.

## 3. Results

This study included 171 female patients with the Apfel simplified score ≥ 2 (82 in the NK1 group and 89 in the ONS group). No significant differences in patient demographics, risk factors for PONV, duration of anesthesia and surgery, blood loss, and fluid volume were observed between the groups (Table 1).

There was no significant difference in the percentage of patients with no vomiting and no rescue (complete response) at 0–2 hours (78 versus 80%, resp.; *P* = 0.852), 0–24 hours (71 versus 67%, resp.; *P* = 0.741), and 0–48 hours (71 versus 67%, resp.; *P* = 0.741) after surgery. However, the incidence of vomiting was significantly lower in the NK1 group compared to the ONS group at 0–2 hours (2 versus 17%, resp.; *P* = 0.0016), 0–24 hours (2 versus 28%, resp.; *P* < 0.0001), and 0–48 hours (2 versus 29%, resp.; *P* < 0.0001) after surgery (Table 2). Where rates of vomiting were adjusted for use of rescue antiemetic, both groups were similar in terms of rescue antiemetic use for the 0–48 h interval. The rescue antiemetic was administered 10 mg metoclopramide when the patients required postoperative rescue antiemetic. No statistically significant differences in PONV, rescue antiemetic use, and nausea score were observed between the groups for any analyzed period (Tables 2 and 3).

## 4. Discussion

This study indicated that fosaprepitant is superior to ondansetron in preventing vomiting 0–2, 0–24, and 0–48 hours after surgery in moderate to high PONV risk patients. No significant differences were observed between fosaprepitant and ondansetron in incidence of PONV, complete response rate, use of rescue antiemetics, and severity of nausea.

Several neurotransmitter receptor systems including dopaminergic, cholinergic, histaminergic, serotonergic (5HT-2 and 5HT-3), and NK1 systems are associated with nausea and vomiting [18]. 5HT-3 receptor antagonists and NK1 receptor antagonists were shown to be effective in preventing CINV. A corticosteroid and a 5HT-3 receptor antagonist are often combined for preventing CINV. Recently, NK1 receptor antagonists, aprepitant and fosaprepitant, have become available and their use has improved the management of CINV [19]. In the 2016 update of MASCC/ESMO consensus guidelines, for prophylaxis of acute and delayed nausea and vomiting induced by chemotherapy, a combination of a 5-HT3 receptor antagonist, dexamethasone, and a NK1 receptor antagonist is recommended [20]. NK1 receptor antagonists are expected to reduce the incidence of CINV.

NK1 receptor antagonist aprepitant was shown to be effective in preventing PONV. Diemunsch et al. [12] suggested that aprepitant was significantly superior to ondansetron in preventing vomiting. Gan et al. [11] showed that aprepitant was as effective in reducing nausea as ondansetron and more effective in preventing vomiting during the first 24 and 48 hours after surgery. Moreover, analysis of data from two clinical trials [13] indicated that aprepitant was superior to ondansetron in preventing PONV.

In this study, we used fosaprepitant, a prodrug of aprepitant. Fosaprepitant is converted to aprepitant within 30 minutes of intravenous administration and blocks NK1 receptors. A dose of 115 mg of fosaprepitant was reported equivalent to 125 mg of aprepitant [10], with intravenous administration of 150 mg of fosaprepitant showing the same efficacy as a 3-day oral aprepitant treatment (125/80/80 mg) [21]. In our previous studies [15–17], we demonstrated the efficacy of fosaprepitant in preventing PONV in patients undergoing general anesthesia compared to ondansetron. This study enrolled moderate to high PONV risk patients and confirmed fosaprepitant efficacy compared to ondansetron. Improved efficacy of fosaprepitant in preventing postoperative vomiting may be attributed to its long half-life.

For moderate to high PONV risk patients, multimodal prevention approaches can reduce the risk of PONV. Consensus guidelines for managing postoperative nausea and vomiting [4] recommend an antiemetic combination of dexamethasone (4 mg) and ondansetron (4 mg). Considering the superior antiemetic effect of fosaprepitant compared to ondansetron in this study, a combination of dexamethasone and fosaprepitant (instead of ondansetron) may be more effective. Moreover, long half-life of fosaprepitant suggests that a single administration of fosaprepitant may be equivalent or more effective than a combination dexamethasone-ondansetron treatment in preventing PONV. Further evaluation of clinical characteristics of fosaprepitant in combination with other antiemetics for PONV prevention is planned.

Certain limitations should be noted. Fosaprepitant has a much longer half-life than ondansetron and, consequently, longer duration of activity, which may account for improved prevention of vomiting. Additionally, as antiemetics are expected to be more effective when administered towards the end of surgery compared to during induction of anesthesia, timing of drug administration could have affected our results.

Moreover, fosaprepitant treatment is relatively expensive, costing US$ 122.5 in Japan. However, as use of antiemetics can reduce complications associated with PONV, preventing prolonged hospital stays, total medical costs may decrease. As we did not evaluate long-term outcomes in this study, it remains unclear whether reduction of postoperative vomiting by fosaprepitant improves postoperative outcomes or provides additional benefits for patients. Further studies are required to evaluate the contribution of fosaprepitant treatment to postoperative outcomes.

## 5. Conclusions

This study demonstrated that NK1 receptor antagonist fosaprepitant was superior to 5HT-3 receptor antagonist ondansetron in preventing vomiting 0–2, 0–24, and 0–48 hours after surgery in moderate to high PONV risk patients. However, no significant differences were observed between fosaprepitant and ondansetron in PONV incidence, complete response, use of rescue antiemetics, and nausea severity at any period analyzed.

## Figures and Tables

**Table 1:** Patient demographics.

	NK1 group	ONS group
Patients	*n* = 82	*n* = 89
Age, years	57 ± 14	56 ± 13
Height, cm	154 ± 8	155 ± 6
Weight, kg	57 ± 11	56 ± 10
ASA PS I/II/III	23/58/1	27/61/1
PONV risk factors		
Nonsmoker (*n*)	80	88
History of motion sickness (*n*)	26	22
Women (*n*)	82	89
Postoperative opioids, *µ*g	470 ± 449	443 ± 451
Duration of anesthesia, min	269 ± 124	280 ± 138
Duration of surgery, min	209 ± 113	219 ± 129
Anesthetics; remifentanil, mg	3.4 ± 2.4	3.2 ± 3.2
Blood loss, ml	321 ± 488	251 ± 360
Fluid volume, ml	2245 ± 1312	2127 ± 1101

Data are presented as means ± SD (or range) or number of patients; NK1 group, i.v. fosaprepitant; ONS group, i.v. ondansetron; PONV, postoperative nausea and vomiting; ASA PS, American Society of Anesthesiologists physical status.

**Table 2:** Postoperative parameters.

	NK1 group, *n* = 82	ONS group, *n* = 89	*P* value
0–2 hours			
PONV	34 (41%)	25 (28%)	0.077
Complete response	64 (78%)	71 (80%)	0.852
Vomiting	2 (2%)^*∗*^	15 (17%)	0.0016
Rescue antiemetic use	18	13	0.238
0–24 hours			
PONV	45 (55%)	44 (49%)	0.647
Complete response	58 (71%)	60 (67%)	0.741
Vomiting	2 (2%)^*∗*^	25 (28%)	<0.0001
Rescue antiemetic use	30	26	0.331
0–48 hours			
PONV	45 (55%)	47 (53%)	0.878
Complete response	58 (71%)	60 (67%)	0.741
Vomiting	2 (2%)^*∗*^	26 (29%)	<0.0001
Rescue antiemetic use	30	29	0.631

Data are presented as number of patients (percentile); NK1 group, i.v. fosaprepitant; ONS group, i.v. ondansetron; PONV, postoperative nausea and vomiting. ^*∗*^*P*<0.05 compared to ONS group.

**Table 3:** Nausea and NRS.

	NK1 group, *n* = 82	ONS group, *n* = 89
0–2 hours		
Severity of nausea (0/1/2/3)	48/15/10/9	65/6/4/14
2–24 hours		
Severity of nausea (0/1/2/3)	62/10/6/4	57/10/10/12
24–48 hours		
Severity of nausea (0/1/2/3)	76/5/1/0	77/6/2/4

Data are presented as number of patients or median (interquartile range); NK1 group, i.v. fosaprepitant; ONS group, i.v. ondansetron; severity of nausea: 0 = absence of nausea, 1 = mild nausea, 2 = moderate nausea, and 3 = severe nausea.
